# Editorial: Multidisciplinary approach to interstitial lung disease associated with systemic rheumatic diseases

**DOI:** 10.3389/fmed.2022.1112872

**Published:** 2022-12-13

**Authors:** Gianluca Sambataro, Stefano Palmucci, Fabrizio Luppi

**Affiliations:** ^1^Regional Referral Centre for Rare Lung Disease, Azienda Ospedaliero-Universitaria Policlinico G. Rodolico-San Marco, University of Catania, Catania, Italy; ^2^Artroreuma S.R.L., Rheumatology Outpatient Clinic, Catania, Italy; ^3^Department of Medical, Surgical Sciences and Advanced Technologies G.F. Ingrassia, Radiology I Unit, University Hospital Policlinico G. Rodolico-San Marco, Catania, Italy; ^4^Respiratory Diseases Unit, San Gerardo Hospital, Monza University of Milano-Bicocca, Milan, Italy

**Keywords:** interstitial lung disease, rheumatoid arthritis, antisynthetase syndrome, systemic sclerosis, idiopathic inflammatory myopathies (IIMs)

The term “Interstitial Lung Disease” (ILD) defines a deposition of extracellular matrix and/or inflammatory cells in the lung interstitium, potentially leading to respiratory failure and death. Among the numerous conditions associated with ILD, autoimmune diseases account for about 20% of ILD diagnoses (1). ILD is a common feature affecting all Connective Tissue Diseases (CTDs) in proportions ranging from 20% for primary Sjögren's Syndrome (pSS) to up to 75% for Idiopathic Inflammatory Myopathies (IIMs), and, when present, is one of the main causes of mortality (2). Despite this, among the validated criteria for CTDs, the sole condition for which ILD is an item for consideration is Systemic Sclerosis (SSc) (3).

This underestimation of the diagnostic value of ILD in CTDs could be due to a physiological selection bias, as patients in which respiratory involvement represents the main, or even sole clinical involvement, may tend to consult pulmonologists rather than rheumatologists. In a respiratory setting, the recognition of rare although specific clinical manifestations (e.g., Gottron's sign) or nuanced features (e.g., inflammatory arthritis or acrocyanosis) could be more challenging. Moreover, despite the fact that some High-Resolution Computed Tomography (HRCT) ILD patterns are generally considered suggestive of specific CTDs (e.g., Non-specific Interstitial Pneumonia, NSIP, for SSc or Lymphocytic Interstitial Pneumonia, LIP, for pSS), almost all of the patterns can be present in each CTD.

Close collaboration between rheumatologists, pulmonologists, and radiologists allows knowledge to be shared between the three figures, with great reciprocal benefits for the management of CTD-ILD patients. For this reason, Multidisciplinary Discussion is currently considered to be the gold standard for the management of all ILDs (4). This collaboration, which improves the recognition of CTD-ILD patients, also allows the enrolment of a population that is more representative of the actual condition, with consequent benefits also for research.

On this Research Topic, we include with great pleasure the systematic review written by Landini et al., in which the authors evaluated the value of HRCT SSc-ILD in predicting a worse outcome. The authors defined a worse outcome as death (due to overall and respiratory causes), need for oxygen supplementation or lung transplant. After evaluating more than 3,500 citations, extensive ILD proved to be an independent predictor of worse outcomes under all of the definitions provided. Other possible fibrotic parameters such as fibrotic extent and reticulation extent were associated with overall mortality, while the presence of honeycombing, the expression of a Usual Interstitial Pneumonia (UIP) pattern, was associated with respiratory mortality. In the study, the authors also report the current state of knowledge on radiological management of SSc-ILD, explaining unfulfilled needs, mainly regarding the radiological fibrotic phenotype. SSc-ILD is classically associated with an NSIP pattern, however elements suggestive of UIP are not uncommon (2). The early recognition of radiological features suggestive of a fibrotic process could have a significant impact on the daily management of these patients, considering the recently discovered possibility of providing treatments potentially able to slow fibrosis.

UIP represents the most common pattern associated with Rheumatoid Arthritis (RA)-ILD. Readers can find a very interesting comprehensive review on this topic, with great practical impact (Laria et al.). In this paper, Laria et al. describe epidemiology, clinical manifestations, risk factors, and potential treatments for all of the potential forms of respiratory involvement associated with RA. The manuscript is enhanced by an explanatory collection of figures regarding RA-ILD and an interesting proposal for the management of the condition. The proposed algorithm is based on both Rheumatology and Pulmonology perspectives, therefore it could be useful to both of the main subsets these patients refer to, explaining management and therapeutic options in light of the current knowledge in literature. This reasonable approach can be further appreciated considering that current severity indexes for RA are almost entirely limited to describing joint damage and the current guidelines for therapeutic management of RA proposed by the European League Against Rheumatism (EULAR) and the American College of Rheumatology do not cite ILD (5–7).

Finally, Wells et al. highlight the crucial role of a Multidisciplinary team involving pneumologists, rheumatologists and radiologists in the diagnosis of Antisynthetase Syndrome (ASSD). ASSD is a CTD included on the IIM spectrum. Despite the fact that a classic triad is well-established for the diagnosis of this condition (ILD, inflammatory arthritis, myositis), validated classification criteria are not currently available (they should be released in 2023 as the fruit of a joint effort between the ACR and EULAR within the CLASS project). The “selection bias” cited in the introduction of this manuscript probably has a significant effect on current knowledge on this condition: in fact, ILD could be the first or even the sole clinical manifestation of the disease, mainly in patients positive for non-anti-Jo1 antisynthetase antibodies (8). These patients also show a worse respiratory prognosis and are at high risk of being misdiagnosed, as non-anti-Jo1 antisynthetase antibodies are not included in the most common commercial kits for Extractable Nuclear Antigens. However, correct diagnosis is crucial to providing appropriate treatment and conducting further studies on this condition. In their interesting review, Wells et al. provide a comprehensive summary of current knowledge on the clinical, serological, and radiological features of ASSD patients, enriching the manuscript with informative images of the muscular and pulmonary involvement typical of this disease. The authors also report on a useful clinical approach that can drive laboratory and instrumental assessment of the main clinical manifestation of the disease.

In conclusion, despite sometimes being difficult to implement in clinical practice, a multidisciplinary approach to CTD-ILD is essential for both clinical and research purposes. We believe that the studies included on this topic could be very useful in supporting the diagnosis and management of the three CTDs (RA, SSc, ASSD) in which ILD is associated with the worst respiratory outcomes.

## Author contributions

The manuscript was written by all authors together and all authors approved its final version.
